# Psychiatric nurse's perceptions of their interactions with people who hear voices: A qualitative systematic review and thematic analysis

**DOI:** 10.1111/jpm.12829

**Published:** 2022-04-08

**Authors:** Anita McCluskey, Chanel Watson, Linda Nugent, Tom O’Connor, Zena Moore, Niall O’Brien, Luke Molloy, Declan Patton

**Affiliations:** ^1^ School of Nursing and Midwifery RCSI University of Medicine and Health Sciences Dublin Ireland; ^2^ Skin Wounds and Trauma Research Centre RCSI University of Medicine and Health Sciences Dublin Ireland; ^3^ RCSI University of Medicine and Health Sciences Dublin Ireland; ^4^ School of Nursing Faculty of Science, Medicine and Health University of Wollongong Wollongong New South Wales Australia

**Keywords:** auditory hallucinations, hearing voices, nurse interactions, health and wellbeing.

## Abstract

**What is known on the subject?:**

- There is no qualitative systematic review of nurses' perceptions of their interactions with people hearing voices. There are some studies exploring the interventions provided by community psychiatric nurses to people hearing voices; these give a sense of what interactions may contain.

**What the paper adds to the existing knowledge?:**

- Nurses across both community and inpatient mental healthcare settings feel uncertain about how to interact with people hearing voices, sometimes feeling like they can do little to help. Their interactions are affected by the workplace culture, education and training and concern for their own safety. Nurses rely on a therapeutic relationship for all interactions.

**What are the implications for practice?:**

- This is an under investigated area of mental healthcare. None the less this qualitative systematic review highlights that nurses are unclear about how to interact with service users hearing voices with the resultant outcome that service users in great distress may only be receiving minimal benefit from their interactions with the nurses caring for them.

**Abstract:**

**Aims and Objectives**

The aim of this qualitative systematic review and thematic analysis was to identify and synthesize results from studies that explored psychiatric nurses' perceptions of their interactions with service users experiencing auditory hallucinations (hearing voices).

**Method**

Qualitative systematic review and thematic analysis.

**Results**

Five studies that met the inclusion criteria were identified. Nurses reported that they felt uncertain about what to do for service users hearing voices, struggled to have interactions with voice hearers, but greatly valued the therapeutic relationship with service users. Nurses also reported that they required more education and training on how to interact effectively with people hearing voices. Finally, various workplace challenges were identified as an important factor mediating nurse interaction with service users hearing voices.

**Discussion**

Existing evidence shows that nurses lack clarity about how they can interact effectively and in a way that helps service users who are hearing voices. Significant barriers that they must overcome in order to be more certain of their role in caring for people hearing voices are difficult to engage service users and workplace challenges that were not conducive to helpful interactions and conversations.

**Implications for Practice**

Nurses caring for people hearing voices require more and better education and training with a view to them becoming more confident and competent when interacting with this service user group. Furthermore, healthy workplace cultures and maintaining a safe environment are necessary for effective caring interactions with people hearing voices.

## 1 INTRODUCTION

Auditory hallucinations (hearing voices) are described as a perceptual involuntary experience that occur without an appropriate external stimuli, (David, 2004; Longden et al., 2011). A recent meta‐analysis concluded that up to one in ten individuals report hearing voices, and that the prevalence of hearing voices across the lifespan decreases with age, ranging from a prevalence in children and adolescents of between 12.7% and 12.4%, reducing in adulthood to 5.8%, and reducing again to a prevalence of 4.5% in elderly people (Maijer et al., 2018). Hearing voices can occur in a range of psychiatric disorders such as schizophrenia, post‐traumatic stress disorder (PTSD) and emotional unstable personality disorder (Laroi et al., 2012). Research has shown that 25% of people that reported hearing voices met the relevant criteria for a psychotic disorder diagnosis (Leed‐Smith & Barkus, 2013).

There are four ways in which hearing voices occur. These include persistent commanding/commenting hallucinations, voices of the individual's own thoughts, non‐verbal hallucinations and broadcasting auditory hallucinations (Wang & Xu, 2013). Voice hearing hallucinations are heterogenous; they can vary from first, second and third person commentary, from transient utterances of a simple sound to single words or full conversations with familiar and unfamiliar voices. They can be complimentary and pleasant, but usually they are unpleasant and insulting (Laroi et al., 2012). These voices can be distressing and threatening towards the individuals that hear them (Nayani & David, 1996), causing disruption in all aspects of their life; socially, occupationally and psychologically (Hayward et al., 2017; Ruddle et al., 2011). It has been reported by Larøi et al. (2019) that a significant cohort of people reports hearing voices with a negative content.

While guidelines outline that people hearing voices should be offered cognitive behaviour therapy for psychosis (CBTp; NICE 2014). CBTp is a talking therapy designed to help people experiencing psychosis to gain better coping strategies for their mental health difficulties (Health Service Executive 2021). Yet these CBTp services are rarely provided to people suffering from psychosis (Royal College of Psychiatrists, 2018), with funding and a lack of expert practitioners blocking the provision of what is considered a resource‐intensive therapy. In the meantime, informal conversations by practitioners such as psychiatric nurses can be valuable and potentially beneficial (Bogen‐Johnston et al., 2019). However, little is known about these interactions, despite the importance of the nurse–service user relationship (Delaney 2018).

According to Coffey and Hewitt (2008), service users hearing voices revealed that they desired more direct engagement with their Community Mental Health Nurses (CMHNs), such as exploring the meaning behind the voices they were hearing. Other evidence also suggests that service users place great value on healthcare providers discussing the voices they hear with them (Coffey et al., 2004; Place et al., 2011; Romme & Esher, 1989; Romme & Morris, 2007). With research indicating that service users welcome nurses engaging with them about their voice hearing experiences, it is important to understand the nuances of these interactions for clinical practice.

Psychiatric nurses represent the largest health professional group across mental healthcare services internationally (Jones & Coffey, 2012). Nurses are the key frontline care providers in most mental healthcare facilities; they are with service users 24 h a day in some care settings and have countless interactions with service users on a daily basis (Niu et al., 2019). In light of this, there is a growing interest in clarifying the exact contribution of nursing care towards the service users' recovery and health (Morris et al., 2014). The purpose of this review will be to explore the evidence on how nurses perceive the interactions they have with service users hearing voices.

## 2 QUESTION

“What are the reported psychiatric nurses' perceptions of their interactions with people who hear voices?”

## 3 AIMS

The aim was to identify and synthesize results from studies that explored nurses' perceptions of their interactions with mental healthcare service users hearing voices, and discuss how nurses could optimize their responses to service users hearing voices.

## 4 METHODOLOGY

This paper reports a qualitative systematic review of studies that explore nurses' perceptions of their interactions with service users experiencing auditory hallucinations. In what is a newly emerging approach to healthcare research, the qualitative systematic review aims to present a comprehensive grasp on the experiences or perceptions of research participants (Stern et al., 2014). Much the same as the more traditional systematic review, a qualitative systematic review intends to produce a high quality and meticulous review of the best available evidence (Aromataris & Pearson, 2014). As qualitative systematic review techniques are still evolving and developing, there are minimal resources available to guide researchers through review process (Butler et al., 2016). However, the Centre for Review and Dissemination's (CRD) (2009) document on systematic reviews was used for guidance to strength the methodological underpinning of this review, aiming to develop a high standard systematic review with supporting methods.

Relevant published literature were searched. The Critical Appraisal Skills Programme (CASP) tool was utilized to assess the quality of the included studies (Long et al., 2020). The CASP tool is considered to be a user friendly choice for beginner researchers, and is endorsed by Cochrane and the World Health Organisation in for use in qualitative synthesis (Hannes & Bennette, 2017). The CASP tool was also originally developed for use within health‐related research (Long et al., 2020), proving to be an appropriate choice for this systematic review. The quality assessment was completed before the synthesis of data as recommended by the CRD (2009). The results of the review were analysed using thematic analysis. The reporting of this systematic review was guided by the standards of the Preferred Reporting Items for Systematic Review and Meta‐Analysis (PRISMA) Statement.

## 5 METHODS

### 5.1 Search strategy

An electronic literature search was conducted with the assistance of a health sciences librarian in October 2021. The search included the following databases: PsychINFO, Medline, CINAHL and the Cochrane Library database. The search terms were psychiatric nurse OR mental health nurse OR psychiatric nursing AND interactions OR interventions OR best practices OR nurse‐patient relations AND auditory hallucinations OR hearing voices OR voice hearing. A manual search of Google Scholar was also conducted with the same search terms to identify other pertinent papers not identified earlier, as per CRD guidance (2009). The literature search identified a total of five published papers suitable for inclusion in the review. There were no qualitative systematic reviews identified on this topic to date.

### 5.2 Inclusion and exclusion criteria

The inclusion and exclusion criteria were as follows:

#### 5.2.1 Inclusion criteria

- Studies focusing on nurses' perspectives of their interactions with people hearing voices
- Qualitative studies
- Studies reported in English

#### 5.2.2 Exclusion criteria

- Studies that did not focus on nurses' perspectives of their inter interactions with people hearing voices.
- Studies published prior to 2000, to ensure the most up to date studies were included.
- Non‐qualitative studies.
- Studies not in the English language.

### 5.3 Screening

**FIGURE 1:**
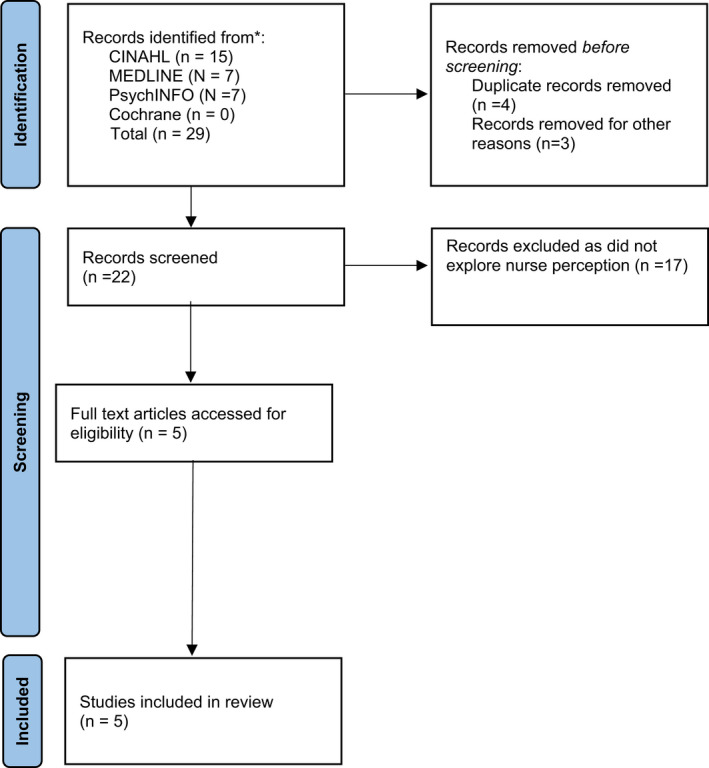
PRISMA flow chart

A total of 29 records were identified before screening. Seventeen papers were excluded after screening as they did not focus on nurses' perceptions of their interactions with people hearing voices. One conference paper, one dissertation and one book were also removed, as were four duplicates. Five papers met the inclusion criteria. The reference lists of the five papers were screened but no further papers were identified. Please see Figure 1.

### 5.4 Thematic analysis

One method suggested for the synthesis of qualitative data is thematic analysis (CRD 2009). Braun and Clarke's (2006) thematic analysis was the method used to synthesize the results in the five selected papers. Braun and Clarke's (2006) approach was utilized as it offers a robust and manageable method for those new to qualitative research (Braun & Clarke, 2006). There were six phases to this method and these were completed manually, to keep the researchers as close and immersed in the data as possible. In phase one, two authors (AMC & DP) read the papers numerous times to gain an in‐depth familiarity with the content of each paper. At this stage, notes were made of possible themes which could be revisited at a later stage, as patterns across papers were sought. Phase two involved the generation of initial codes from the data; this was done independently by the same two authors. Coding is essential as it involves grouping meaningful data into groups (Tuckett, 2005). In phase three, the different individual codes were placed into broader themes, ultimately creating an overarching theme. Themes continued to be refined and reviewed in phase four by the two reviewers with any disagreement resolved by discussion, and if needed consultation by a third author (CW). In phase five, themes were defined, Braun and Clarke (2006) describe this as “determining the essence of the theme.” Phase six is the final phase of the thematic analysis, and this involved the commencement of this paper.

## 6 RESULTS

### 6.1 Overview of studies

Study characteristics are outlined in Table 1. The review included three studies conducted in the United Kingdom (Bogen‐Johnston et al., 2019; Coffey & Hewitt, 2008; McMullan et al., 2018), one in Australia (White et al., 2019) and one in the Republic of Ireland (McCluskey & De Vries, 2020). Combined, the overall sample was 50 nurses, with 26 practising in the community and the remaining 24 practising in in‐patient psychiatric units. In total, 26 of the sample participants were female and 15 were male. Bogen‐Johnston et al. (2019) did not specify the gender of their 9 participants to protect their confidentiality. The five studies all used a qualitative methodology with an interview design.

**TABLE 1:** Study characteristics

Author and year	McCluskey and DeVries (2020)	Bogen‐Johnston et al. (2020)	White et al. (2019)	McMullan et al. (2018)	Coffey & Hewitt (2008)
Aim	Explored psychiatric nurse's perspectives on their care for people who hear voices	To investigate how EIP practitioners work with service users who hear voices	Exploration of early career nurses' experiences when working with VH	Exploration of experiences of staff working with VH in an acute unit	Exploring CMHNs and services views on responses to hearing voices
Methods	Semi‐structured interviews	Semi‐structured interviews	Individual interviews	Individual interviews	Individual interviews
Participants	16 acute unit psychiatric nurses	2 psychiatric nurses (out of 10 participants)	9 psychiatric nurses (3 inpatient, 6 community settings)	3 psychiatric nurses, 5 mental healthcare workers	20 CMHNs. 20 service users
Key findings	Nurses saw great value in establishing and maintaining a strong therapeutic relationship with services as a pre‐condition to any other interventions they provided. The overreliance on medication and lack of structure /focus to interventions were concerning to nurses	Psychiatric nurse participants were supportive of the idea of talking and exploring the voices, but felt uncertain in their ability to do this effectively with service users, despite previous training in this area	Nurses faced challenges when responding to the distress of service users. Nurses felt uncertain and fearful about engaging in discussion about the voices that services users heard, and fearful about the consequences of these discussions. Nurses also felt that they were underprepared regarding their education and training, also citing that some of the skills they had learned in their training was not applicable to an in‐patient setting	Findings in this study highlighted nurses feeling powerless and helplessness at times regarding reducing distress for people hearing voices. Nurses experienced performance anxiety and self‐doubt about their ability to assist this cohort	Findings highlighted differing views between nurses and service users, with service users desiring additional and alternative interventions from nurses. Nurses understood the potential benefit of discussing voices with service users but felt restricted in their ability to do this, they also felt they provided considered and titrated interventions that all reflected the personal circumstances of the service user

### 6.2 Quality appraisal

The CASP (2020) checklist was used to quality appraise the five papers, see Table 2. Overall the CASP checklists identified a high standard of research in all the reviewed papers, demonstrating strengths such as adequate reflexivity, consideration for the relationships between participants and researchers and justified study design and methodologies. Ratings of “yes,” “no,” and “can't tell” were used to signal whether the ten items on the tool were exhibited (CASP 2020). The CASP checklists were carried out independently by two reviewers (AM and DP). When disputes arose, they were resolved through discussion to reach consensus.

**TABLE 2:** Critical appraisal skills programme

McCluskey and DeVries (2020)	Bogen‐Johnston et al. (2020)	White et al. (2019)	McMullan et al. (2018)	Coffey & Hewitt (2008)
The CASP checklist reflected an appropriate methodology and study design, with due regard given to ethical issues and confidentiality. While it is limited, there is reference to some reflexive practices, that is a reflective journal	The CASP checklist reflected a justified research study design, methodology and recruitment procedure, with due attention paid to the dynamic between the researcher and the participants. Ethical approval was also granted	The CASP checklist reflected strong evidence for all sections including study design, methodology, reflexive approach and analysis	The CASP checklist reflected strong evidence for all sections, including reflexivity, data collection, analysis and design	The CASP checklist reflected a strong qualitative paper demonstrating an appropriate study design, methodology and recruitment strategy, with relevant ethical and confidentiality issues addressed. No evidence of a reflexive approach during the study

### 6.3 Thematic synthesis

Five themes were identified; these were as follows: (1). Difficult to engage; (2). Therapeutic relationship; (3) workplace challenges; (4) uncertainty and self‐doubt; and (5). education skills and training. Table 3 provides details on which papers contributed to which themes.

1. Difficult to engage

**TABLE 3:** Papers contribution to themes

Paper	Themes				
	Difficult to engage	Therapeutic relationship	Workplace challenges	Uncertainty and self doubt	Education skills and training
Bogen‐Johnston et al. (2020)	✓	✗	✗	✓	✓
McCluskey and DeVries (2020)	✓	✓	✗	✓	✓
White et al. (2019)	✓	✓	✓	✓	✓
McMullan et al. (2018)	✓	✓	✓	✓	✗
Coffey & Hewitt (2008)	✗	✗	✓	✓	✓

It appears that nurses faced a challenge when initiating an interaction with service users who were hearing voices. While most nurses showed a willingness to engage with the service user and sit down and have a conversation with them surrounding their voice hearing, they also perceived having this interaction as a challenging task. One nurse participant in Bogen‐Johnston et al. (2019) paper reported that

using our engagement skills and that to try and tease out what is going on. But cert, even with some that I see, I've been seeing for two or three years, they don't like to talk about it even though we can sense they're hearing voices from their looking away or responding.

The notion that nursing staff must “tease out” details and discussion around auditory hallucinations highlights that nurses perceive service user's reluctance to engage about their voices as a barrier to their interactions on the topic. Participants in the McCluskey and De Vries (2020) study also referenced the perceived difficulty in engaging and interacting with service users about the voices they heard. Participants spoke of service users potentially feeling uncomfortable during these interactions, and other symptoms playing a role in their limited engagement with nurses, with one participant citing that “Nine times out of ten persons who are experiencing voices might be paranoid, so for you to ask them what are you hearing isn't going to work.” It would appear that it is challenging for nurses to talk to service users who are hearing voices while also deeply paranoid.

Service users not willing to have an interaction with nurses or wanting to divulge details of the voices they are hearing were also experienced by nurse participants in White et al. (2019) with nurses noticing that “they will see you coming, and zip up, and they don't want you to know what's going on.” Another participant in this study described the extreme end of service users being difficult to interact with, citing the potential for service users becoming violent and aggressive, “they start being abusive, physically aggressive or trying to push, trying to kick, trying to punch the wall or breaking things, throwing chairs, things like that.” It is clear that nurses perceive their interactions as somewhat hindered by service users declining to engage, be it for various reasons. Again, this was brought to light by a nurse participant involved in McMullan et al. (2018) who said,

I guess when you… it's… people that hear voices and they might be experiencing them really badly and…. they're just really… loud, they experience them as loud, and you notice that when you're trying to talk to them… ‘oh you're hearing voices?’ and you try and engage them in some sort of distraction and… coping strategies or something. And you can't quite get… you can't quite get to them because they're in that stage where they can't… I guess they need medication and it's difficult to help them then.

In this case, the voices themselves proved to be an obstructive factor for nurses looking to interact with service users hearing voices, delaying and perhaps preventing a potentially useful and comforting interaction for the service user.

2. Therapeutic relationship

The importance of the therapeutic relationship with service users hearing voices was another prominent theme. Developing a trusting relationship, where the service user felt comfortable in sharing their thoughts and emotions regarding their voices during an interaction with nurses was perceived as essential (White et al., 2019), “building rapport and a relationship…if somebody has a good rapport with you, […] they're much more willing to share their vulnerabilities.” This short extract articulates that without a good working alliance with service users, they may not share their true thoughts or experiences, such as their voice hearing. Developing a good rapport with serve users was deemed an essential undertaking for the delivery of optimal nursing care, and if this was not achieved it could hinder all further interactions, with one participant in McCluskey and De Vries (2020) saying,

You're building a rapport with the patient from day one. If you get off to a bad start or they have ideas about you, if you don't address that straight away you could have awful altercations throughout the person's care. They might not feel comfortable coming to you.

This paper found that overall, nurse participants stove to develop a strong interactional relationship with service users at the outset of their care.

Nurse participants across the selected studies valued getting to know service users and developing a strong helping relationship with them; they perceived this very important when it came to having interactions with service users about their voices and other health issues. A good rapport with service users had the potential to help overcome any underlying paranoia or trust issues with staff, as described in the following extract from McCluskey and De Vries (2020),

before I do all that, it's important and imperative that you develop a therapeutic relationship with the patient so that they feel comfortable in discussing any problems that they might be having. Nine times out of ten persons who are experiencing voices might be paranoid, so for you to ask them what are you hearing isn't going to work, so that's why it's important that you develop the relationship with them before you discuss it with them.

McMullan et al. (2018) echoed the sentiment that nurses perceive a therapeutic relationship as the focal point of any interactions with service users hearing voices, with one participant referencing that if a service user trusts their nurse, they will continue to return to that nurse allowing for regular interactions in a perceived safe relationship,

if you build… someone's got to trust you to start talking about those kind of things [voice hearing experiences] so then you build up this rapport with someone. And they remember that, if they come back in again, they'll… they remember you as that person and… instantly feel more… safe around you and not as anxious.

Nurse participants believe that building a strong rapport will enable the service user to engage in more interactions about their voices with staff, ensuring support on a regular basis and when they require it. While nurse participants in three of the reviewed studies were emphatic about the prerequisite of a solid therapeutic relationship for their interactions with service users hearing voices, two studies (Bogen‐Johnston et al., 2019, Coffey & Hewitt, 2008) did not reference this working relationship.

3. Workplace challenges

Another theme that emerged from the data was the perceived challenges that presented themselves when working in a mental health setting, and how these challenges impacted on the interactions between nurses and people who hear voices. McMullan et al. (2018) discovered that nurses felt nervous about providing care and the subsequent interactions with service users hearing voices because of how other staff may perceive them and their approach to and within these conversations, with a particular fear that they may be seen as incompetent by others. One nurse in White et al.'s (2019) study also cited that other nurses' disapproval can be an obstacle in fulfilling the goal of establishing a therapeutic relationship with service users hearing voices.

Another workplace issue that infringed on the interactions between nurses and service users hearing voices was the potential risk to various stakeholders' safety. Promoting the safety of the person hearing voices, other service users and the staff involved materialized in two of the three studies reviewed. White et al. (2019) highlighted that nurses were concerned with their own safety and had a fear of being assaulted when interacting with service users hearing voices, as some had experienced violence in mental healthcare settings in the past. Inevitably, this made it difficult for nurses to interact with service users in a comfortable way, as they felt their safety could potentially be compromised.

The results in McMullan et al. (2018) also highlighted the safety risk that comes with caring for people hearing voices and that this can influence the interactions with patients. Nurses in this study also described a prevailing sense of worry about working in a setting that can be unpredictable and unsafe at times, feeling like they were constantly on “tenterhooks.” Only one nurse in Coffey and Hewitt's (2008) alluded to risk and safety issues when interacting with people hearing voices. This participant raised the issues regarding nurses having interactions with service users and during these conversations helping service users to confront their voices, offering that this may be potentially dangerous and not appropriate for certain people.

4. Uncertainty and self doubt

Self‐doubt and uncertainty over how exactly to help people hearing voices featured heavily in three studies. Participants in McMullan et al. (2018) reminisced on times when they questioned whether they were able to do their job or not, experiencing the feeling of incompetence and uncertainty about their ability as a nurse when it came to interacting with service users hearing voices,

What am I doing? What am I achieving here? Don't [know] what you'd call that. Well you think, “Is it helping you [a voice hearer] coming up to me and talking to me all the time, is it worth having a chat to somebody else, getting a different view point?

Another participant in McMullan et al. (2018) paper describes wanting to do their best for their patient, but nervous about causing further upset and distress, “you don't want to do something wrong, you want to do the best for the patient, you don't want to say the wrong thing because you don't want to escalate something,” this participant oscillating between wanting to help but unsure how to not cause harm in the process.

Performance anxiety when caring for people hearing voices further fed into the notion of helplessness that was mentioned by nurses, as they felt they were unable to help people in their care. A nurse participant in Bogen‐Johnston et al. (2019) echoed the uncertainty and self‐doubt felt by other participants, citing that he felt confident about the utility of discussing voices with service users, but less confident about facilitating these conversations, worrying that he was not competent in delivering voice‐related therapy. Participants in McCluskey and De Vries (2020) also showed signs of discomfort and embarrassment when disclosing that they felt they were at times inadequate and ineffective when providing care to service users hearing voices, with one participant citing that embarrassment when they felt they had no answers for patients hearing voices,

I suppose nurses when they feel helpless they become burnt out because they don't know what to be recommending themselves. It's embarrassing when you don't have the answer, the patient struggles because they are not getting the proper intervention.

Nurses that spoke to Coffey and Hewitt (2008) recounted that they also felt uncertain about how to assist service users in opening a dialogue about their voices. Some felt that they could possibly make the situation worse and cause more harm and distress to the. Nurses commonly reported not knowing what to do and being “unsure of how to respond,” citing “I don't know what else to do,” when service users became distressed by their voices. When asked to describe their interactions with people when they complained of hearing voices, the participants had great difficulty in doing so, instead sharing their perceived lack of ability to help or a lack of awareness that they could help at all (White et al., 2019), with one participant reporting that bar distraction techniques they were unsure of what else they could offer to this population, “Apart from distracting them from the voices or letting them be able to live with the voices – I don't know what else, as a nurse I can do.”

This self‐doubt and uncertainty was offset by some participants in Coffey and Hewitt’s (2008) study; when asked about exploring the meaning of the voices with service users, some participants dismissed the idea that there was any relevant meaning behind such experiences and done so with surprising assurance and confidence, a notable departure from the hesitation and self‐doubt previously identified by participants in the other papers.

5. Education skills and training

Nurses referred to not having the adequate range of interventions to effectively care for a person hearing voices (McCluskey & De Vries's, 2020). Some participants admitted that they considered their primary degree as unsatisfactory and deficient in this respect, leaving them to interact with service users hearing voices in an unstructured manner with one participant stating that “I don't feel in this area we got any training that you know we can implement structured plans,” another recently qualified participant cited similar dissatisfaction with their training, “No there's limited interventions and I'm not long qualified and I still wouldn't feel I've been taught a lot in college.” A third participant was blunt in their summary of their acquired skillset and training for helping people that were hearing voices, “We've minimal interventions and training on what to actually do when we are dealing with auditory hallucinations.”

It was noted by McCluskey and De Vries (2020), that none of the participants in their study mentioned or referenced receiving any additional in‐service or onsite training in caring for people hearing voices. As a result, perhaps of the perceived lack of training and skills around hearing voices, nurses were having interactions that were “ad ‐hoc conversations.”

White et al. (2019) report that all participants in their study believed they did not have the adequate knowledge and skills required for caring for people that are hearing voices, with only one participant being able to specify any specialized skills that were expected of nurses working with voice hearers. One participant elucidated the lack of clear structure for working with people hearing voices, a lack of opportunity for learning on the job and the necessity for self‐directed learning,

when we have someone that presents hearing voices, there's no real structure in our role as to specifically, what we do, and so we have to do a lot of our training for ourselves. (White et al., 2019)

Yet in one circumstance (Bogen‐Johnston et al. 2020), even with additional training in cognitive behavioural therapy, one participant reported that he remained uncertain of his ability to have voice‐related conversations with service users,

I feel confident it's a good thing to talk about the voices and explore them, but a little less confident of doing that in, in the structured way that I've seen or read about. I don't feel as confident that, I don't know if that's me as a clinician or, or whether just not as convinced by that evidence for the voices … I'd probably say it's something I don't feel too skilled in and feel slightly fearful of (breathes in) I suppose I do have a slight fear of making them [the voices] worse for people.

This suggests that further support and follow‐up after training may assist in the application of what is learned at training, and that additional training alone may not be sufficient (Bogen‐Johnston et al., 2019).

Coffey and Hewitt's (2008) exploration of community mental health nurses identified how past nurse training and education did not focus on exploring or delving into the meaning of the voices heard by service users, a traditional approach of avoiding conversations about the voices appears to be dominant in the education of the nurse participants in this particular study, with one participant citing that “my training was definitely you don't talk about the voices.”

## 7 DISCUSSION

This review has found that nurses greatly value therapeutic relationships, mirroring what has been written previously, with some authors going as far as to describe the therapeutic relationship as the “cornerstone” of mental health practice (Delaney et al., 2017; McAllister et al., 2014). Active listening, showing empathy and understanding the service users unique experience are all essential to the development of a therapeutic relationship, which in turn is pivotal to recovery orientated mental health care (Gerace et al., 2018; McAllister et al., 2019). The aforementioned traits were also some of the most desirable qualities of psychiatric nurses articulated by service users (Horgan et al., 2020), further highlighting the significance of the therapeutic relationship. The therapeutic alliance is not only valuable as an entity itself, but this indispensable relationship can improve the effectiveness of other nursing interventions across different care settings (Moreno‐ Poyato & Rodriguez‐Noguerira, 2021), thus having positive impact on clinical outcomes (Norcross & Lambert, 2011).

This review also found that nurses' engagement with service users hearing voices must be understood within the challenging context of managerial demands (Kingston & Greenwood, 2020) and an unpredictable environment (Cleary, 2012). Nurses across the five selected studies spoke of being unable to interact effectively with some service users due to their perceived distrust and paranoia, meaning some service users missed out on the helpful interactions fostered by the therapeutic relationship with nurses. Farooq et al. (2020) agree with this, suggesting that nurses will face challenges when they encounter service users that are not willing to engage with them. Nurses also said that that some service users were unwilling to sit and interact with them. They may have been suspicious of nurses due to their mental illness, or perhaps embarrassed at disclosing their voice hearing experience, not an unusual observation (Henderson et al., 2020). It has also been said by Molin et al. (2016) that the obstruction or absence of such interactions with nursing staff can reinforce the perception of a stigmatizing environment and unclear content of care. Missing out on these potentially beneficial interactions and subsequent working relationship may be damaging to people hearing voices.

Evidence to‐date suggests that nurses are struggling with therapeutic engagement (McAllister & McCrae, 2017; McKeown, 2015), due to non‐engagement and more wide‐ranging issues such as time constraints which limit the amount of time nurses can spend interacting with service users (Harris and Panozzo, 2019) and an increase in administration (Seed et al., 2010; Ward & Cowman, 2007). As a result nurses may use custodial methods of care to maintain safety and to manage service users that present with challenging behaviour, custodial methods of care can then become a barrier to effective engagement (Cutcliffe et al., 2015; McAllister et al., 2021). The evidence suggests that there are potential solutions to these difficulties. According to McKeown et al. (2019), adequate staff levels can reduce the use of custodial methods of care. Some studies have also examined the utility of protected engagement time (Edwards, 2008; Thomson & Hamilton, 2012), but with little success (McCrae, 2014). There is a universal acceptance that therapeutic engagement should be a priority, but there remains no practical or theoretically sound solution to facilitate it successfully and consistently. Thus, a solution is urgently required, as the efficacy of psychiatric nursing is dependent on nurse and service user engagement (Browne & Cashin, 2012, McKeown et al., 2017).

It has long been suggested that nursing has been dominated by a medical approach to care (Roberts et al., 2009). However, psychiatric nursing has undergone a radical transformation in the recent past catapulting the profession to high levels of professionalism and autonomy with advancements including the introduction of degree level education, the development of more advanced and specialized roles within practice (ONMSD, 2012), and nurses being authorized to prescribe medication (Maier, 2019), within their scope of practice. Despite the progress of the profession and the opportunity for growing autonomy in emerging roles, the studies included in this review generated some insights into how nurses believe they did not have the appropriate or adequate skill set required to care for people hearing voices. Nurses are essential to the provision of high‐quality care for people experiencing mental health issues; therefore, high levels of competency are pivotal to this (Happell et al., 2019). In an exploratory mixed method study to identify nursing skills competencies, Cusack et al. (2017) cited that participants reported that the opportunity for education and training was essential. In this review, White et al.'s (2019) declaration that none of the participants believed that they had suitable knowledge and skills to care for people hearing voices highlights the importance of more nurse preparation in this area.

A study by England (2007) revealed that psychiatric nurses' understanding and assessments of voice hearing were limited, aligning this to inadequate clinical experience and knowledge of the voice hearing experience. Innovative educational tools such as voice simulation exercises may assist in improving knowledge and understanding in this area as they provide complex but risk‐free learning to aid the comprehension, knowledge and competency of nursing students (Orr et al., 2013). More importantly, research demonstrates that implemented simulated learning within nursing curricula is associated with increased clinical skills and competence (Alinier et al., 2006).

However, two recent papers suggest that after undergraduate nurse training, nurses still felt underprepared and desired further training (White et al., 2019, Bogen‐Johnston et al., 2019). This shows that there is a need to develop and form appropriate tools for learning that will enable psychiatric nurses to grow their scope of practice. Psychiatric nurses make up the largest cohort of professionals in the mental healthcare workforce. Therefore, there is an explicit advantage in guaranteeing that these nurses have a robust set of therapeutic skills directed towards meeting the needs of service users and their distress (Horgan et al., 2020).

In terms of uncertainty, participants felt they were not equipped with the adequate training and education to feel confident and assured when caring for people hearing voices. Cusack et al. (2017) maintain that due to the restructuring of mental health policy to a more recovery focus, psychiatric nurses' roles require clarification. Bladon (2018) concurs, articulating that psychiatric nursing has undergone radical change since its emergence in the last century. A lack of coherence and vague descriptions of the precise role of psychiatric nurses is afflicting the profession, with a specific definition of their role remaining elusive (McAllister & McCrae, 2017). Compounded by a stark contrast between a dominant biomedical model and the desired recovery‐based parameters that nurses are trying to work by (Myklebust et al., 2018), psychiatric nurses may be adrift somewhere in between. Psychiatric nurses in a recent study described intertwining biological, sociological and psychological roles based on service user needs (Hurley & Lakeman, 2021). Perhaps if the core role and skills of psychiatric nursing were identified, appropriate education would then be created and made available (Grant, 2006), thus reducing nurse's experience of such self‐doubt and uncertainty.

Another aspect of psychiatric nursing that may increase clinical capability and capacity to provide meaningful interactions is a healthy workplace culture. A workplace culture reflects its values, beliefs and norms. It is essential that staff have mutual values and beliefs as they work together in order to achieve a set of shared goals (Kurjenluoma et al., 2017). An unhealthy or unsupportive workplace culture can have an impact on employee empowerment, interdisciplinary relations, the use of evidence‐based practice and the alignment with the organizational strategic direction (Moss et al.2017).

This review reported an unsupportive and judgemental workplace for nurses. Aschebrock et al. (2003) reported a similar hesitation by nurses to utilize certain approaches for hearing voices specifically due to the fear of ridicule by other professionals. This type of feared reaction has been described as “horizontal violence.” Horizontal violence involves nonphysical intergroup conflict that is expressed in overt and covert hostile behaviours (Bloom, 2019). Furthermore, Randle (2003) relates that downgrading another person's work can have an effect both self‐esteem and job satisfaction. Another theme that emerged from this review that threatens the performance of healthcare organizations was the perceived risk to the personal safety of nursing staff while caring for people that hear voices. Nurses are in close and constant contact with service users; this results in countless interactions; thus, nurses are at risk of encountering incidents of violence (Niu et al., 2019), moreso then other healthcare providers (Arnetz et al., 2014).

Psychiatric nurses experiencing violence or a threat of physical violence against them can result in negative outcomes for both themselves and the healthcare organization they are employed by. Understandably, nurses can experience physical injuries, but they can also face psychological consequences, including fear, anxiety, guilt, and shame and an increased intent to leave the organization (Stevenson et al., 2015), as well as low morale and reduced quality of care (Campbell et al., 2011).

Participants in this review voiced feeling fear and apprehensiveness when providing interventions for people hearing voices. In a recent studying exploring the experiences of newly qualified nurses beginning their career in mental health units in South Africa, participants also described feeling fearful when faced with the realities of a psychiatric unit (Mabala et al., 2019). It has been suggested by Happell et al. (2019) that overcoming fear and anxiety is undeniably necessary when it comes to the provision of high‐quality care for people experiencing mental health issues. Considering both the negative impacts on nursing staff and patient care, it is crucial that healthcare leaders act to prevent these incidents of violence, reducing the fear and anxiety that is experienced by psychiatric nurses. To achieve this, healthcare organizations need to provide adequate staff, training and assistive devices to reduce these incidents (Niu et al., 2019).

## 8 STRENGTHS AND LIMITATIONS

While ascertaining the nursing perspective can be considered a strength of this paper, it is important to note that this may not be consistent with the service user focused evidence, so therefore findings should be interpreted with a degree of caution. To complete the thematic analysis, the approach by Braun and Clarke (2006) was used. This is a new way of completing systematic review thematic analysis and may require future studies to solidify its reliability as an approach. This review focused solely on qualitative papers, a future review may wish to broaden the search to include research from other types of studies.

## 9 CONCLUSION

Psychiatric nurses perceive several barriers to interacting with service users hearing voices, including a perceived lack of adequate education and skills, workplace challenges and an uncertainty of how to help this cohort of people. Nurses also reported that it was difficult to engage with people hearing voices; however, they were acutely aware of the importance and benefit of establishing and maintaining a therapeutic relationship with voice hearers. Further systematic reviews are needed from the service user perspective, and more primary research is needed into the area generally.

## 10 IMPLICATIONS FOR PRACTICE

There are a number of implications for mental health nursing practice. First, there is a need for further research to be conducted with nurses and their interactions with people that are hearing voices. This systematic review included only 5 papers such was the dearth in evidence in this area. Second, further research is needed into what supports psychiatric nurses require to engage with service users that hear voices. Third, more research should be aimed at investigating what service users deem to be helpful interactions. Finally, nursing management and policy makers should be cognisant of providing and promoting a safe and supportive environment for psychiatric nurses to work confidently and efficiently, providing the highest standard of care possible.

## AUTHOR CONTRIBUTIONS

Anita McCluskey: Anita contributed to all aspects of the systematic review, including conducting the electronic search, completing the thematic analysis and compiling the results. Chanel Watson assisted in all aspects of the systematic review, offering specific expertise and feedback regarding the thematic analysis of the systematic review, providing feedback on all stages of the analysis. Linda Nugent consulted on all drafts of the systematic review, offering feedback on all versions. Tom O’Connor contributed to the discussion and findings section of the review, providing feedback and advice. Zena Moore provided feedback in the early drafts of the systematic review, and contributed to the presentation of the paper. Niall O'Brien Niall was the librarian who assisted in the completion of the search. The assistance of a librarian is written about in the paper. Luke Molloy reviewed, revised and provided feedback on early versions of the paper. Declan Patton assisted in the search strategy of the review, and contributed feedback and advice in this section.

## Data Availability

Data availability is not applicable to this article as no new data were created or analysed in this study.
